# Teamwork and mental workload in postsurgical pediatric patient handovers: Prospective effect evaluation of an improvement intervention for OR-PICU patient transitions

**DOI:** 10.1007/s00431-023-05241-4

**Published:** 2023-10-11

**Authors:** Matthias Weigl, Martina Heinrich, Julia Rivas, Florian Bergmann, Matthias Kurz, Clemens Silbereisen, Hans-Juergen Dieterich, Beate Kleine, Susanne Riek, Martin Olivieri, Florian Hoffmann, Victoria Lieftüchter

**Affiliations:** 1 Institute for Patient Safety, University Hospital, Bonn, 53127 Germany; 2 Institute and Clinic for Occupational, Social and Environmental Medicine, LMU University Hospital, LMU Munich, Munich, Germany; 3 Department of Pediatric Surgery, Dr. von Hauner Children’s Hospital, LMU University Hospital, LMU Munich, Munich, Germany; 4 Department of Anesthesiology, LMU University Hospital Munich, LMU Munich, Munich, Germany; 5 Department of Pediatrics, Dr. von Hauner Children’s Hospital, LMU University Hospital, LMU Munich, Munich, Germany

**Keywords:** Patient handover, Intervention, Teamwork, Workload, Operating room, Intensive care unit, Pediatric care, Checklist

## Abstract

Postsurgical handover of pediatric patients from operating rooms (OR) to pediatric intensive care units (PICU) is a critical step. This transition is susceptible to errors and inefficiencies particularly if poor multidisciplinary teamwork occurs. Despite wide adoption of standardized handover interventions, comprehensive investigations into joint effects for patient care and provider outcomes are scarce. We aimed to improve OR-PICU handovers quality and sought to evaluate the intervention with particular attention to patient care effects and provider outcomes. A prospective, before-after-study design with an interrupted-series and a multi-source, mixed-methods evaluation approach was established. Drawing upon a participative plan-do-study-act approach, a standardized, checklist-based handover process was designed and implemented. For effect assessments, we observed OR-PICU handovers on site (pre implementation: *n* = 31, post: *n* = 30), respectively, with standardized expert observation and provider self-report tools (*n* = 111, *n* = 110). Setting was a tertiary Pediatric University Hospital. Supplementary qualitative, semi-structured interviews were conducted, and a general inductive content analysis approach was used to identify key facilitators and barriers on implementation. Improvement efforts focused on stepwise implementation of (1) standardized handover process and (2) a checklist for multi-professional OR-PICU handover communication. We observed significant increases in team and patient setup (pre: 79.3%, post: 98.6%, *p* < .01), enhanced team engagement (pre: 50%, post: 81.7%, *p* < .01), and comprehensive information transfer by the anesthesia sub-team (pre: 78.6%, post: 87.3%, *p* < .01). Expert-rated teamwork outcomes were consistently higher, yet self-reported teamwork did not change over time. Provider perceived stress and disruptions did not change, mental workload tended to decrease over time (pre: *M* = 3.2, post: 2.9, *p* = .08). Comprehensiveness of post-operative patient information reported by PICU physician increased significantly: pre: 65.9%, post: 76.2%, *p* < .05. After implementation, providers acknowledged the importance of standardized handover practices and associated benefits for facilitation of information transfer and comprehensiveness. Among reported barriers were obstacles during implementation as well as insufficient consideration of professionals’ individual workflow after surgery.

*Conclusion*: A multidisciplinary intervention for postsurgical pediatric patient handovers was associated with improved expert-rated teamwork and fewer omissions of key patient information over time. Inconsistent results were obtained for provider-rated mental workload and teamwork outcomes. The findings contribute to a better understanding concerning the interplay of teamwork and provider cognitions in the course of establishing safe patient transitions in pediatric care.

**Table:** 

**What is Known:** *• Transfer of critically ill children conveys significant challenges for interprofessional communication and teamwork. Prospective research into interventions for safe and efficient handover practices of OR PICU patient transitions is necessary.* *• Checklists are assumed to facilitate cognitive load among providers in acute clinical environments.*	
**What is New:** *• A standardized, checklist-based handover intervention was associated with improvements in team set-up and information transfer. Provider outcomes such as mental workload and stress did not change over time.* *• The combination of teamwork and provider assessments allows a more nuanced understanding of implementation barriers and sustainable effects in course of OR-PICU handover interventions.*

**Supplementary Information:**

The online version contains supplementary material available at 10.1007/s00431-023-05241-4.

## Background

The transition of critically ill children from the operating room (OR) to the pediatric intensive care unit (PICU) is a sensitive task with particular risks to patient safety and quality of care. This transition has been shown to be a challenging and complex teamwork task to ensure smooth transfer of intra and inter professional care responsibilities [1]. OR-PICU patient transitions require handoffs to exchange specific information and transmit responsibilities of care and ensuing treatment.

OR to PICU patient transfers convey complex exchanges of patient information concerning patient’s identification, case characteristics, surgical procedure, and acute or upcoming needs for postoperative care. Despite being critical for patient safety, due to a variety of issues PICU handovers can often get derailed. Available investigations on transfer practices in pediatric care revealed various vulnerabilities such as information loss and misunderstandings with potential patient harm [2–7]. To this end, well-designed and evidence-based interventions to improve OR-PICU handover practices are necessary [8].

The literature on patient handovers in pediatric care settings suggests that substantial improvements can be achieved through systematic interventions [9–18]. Particularly, if improvement efforts are adapted to local context and demands as well as if structured handover tools or standardized measures are implemented [10–12, 19]. Respective evaluation studies show that improvements in terms of transmitted information as well as handover quality can be obtained from the perspective of involved physicians and nurses [u.a. 13, 20]. Moreover, relevant patient outcomes are positively improved as well [11, 14, 17, 18, 21, 22]. Nonetheless, several shortcomings remain in the current literature base on OR-PICU improvement efforts.

First, one key attempt to promote effective handover practices is the consideration of provider mental workload and stress. The consideration of cognitive load theory may help to understand the specific challenges and needs in the course of care transitions and to include cognitive system factors [23]. Therein, three key mental load factors have been suggested with close link to the quality and process of the handover: (1) to titrate the intrinsic mental task load in course of the handover (e.g., use of checklists), (2) to reduce the extraneous load (e.g., eliminate interruptions/distractions during the transition), and (3) to optimize the germane load (e.g., enable co-construction) [23].

Secondly, improvement efforts of patient handovers in hospital practice often take place in complex multi-professional work and care systems with various effects for tasks, collaborations, and processes [24]. Thus, inclusive and provider-oriented improvements are necessary with comprehensive effect evaluations that account for different stakeholder perspectives of physicians and nurses [25–27]. To this end, evaluation across all involved professionals is necessary to obtain a comprehensive picture of inter-professional collaboration and transitional safety.

Third, prospective evaluations with quantitative and qualitative outcomes are necessary to determine the process and overall effects of the handover improvement efforts. Implementation science suggests to augment quantitative effect measures with qualitative information on facilitators and barriers of the intervention process, actual realization of the intended changes, and elicit lessons for similar projects in hospital practice [28].

## Objective

We therefore sought to identify prospective effects of the implementation of a multi-component intervention on OR-PICU handovers. Specifically, our prospective study aimed to.

1. determine changes in information transfer setup and transmitted information,
2. determine changes in provider- as well as expert-rated handover teamwork,
3. determine change in provider mental workload,
4. determine changes in essential information transfer relevant to PICU patient care; as well as
5. describe facilitators and barriers for successful implementation.

## Methods

### Design and OR-PICU setting

A prospective before-and-after study with an interrupted-time series design was established for prospective evaluation of potential changes between baseline and follow-up [29]. A multi-methods assessment of structured expert observations of OR-PICU patient handovers, standardized provider self-reports, clinician reports, as well as stakeholder interviews were established. The baseline assessments were conducted in August 2017 to April 2018. The intervention started in June 2018 and ran until October 2020. Follow-up assessment period was from April 2021 until May 2022.

Setting was a tertiary university children’s hospital in Germany (190 patient beds). This academic hospital covers all fields of pediatric care, provides highly specialized services, and it is one of the largest pediatric hospitals in Germany with around 41,500 outpatients and 6000 inpatients per year.

Ethical approval was granted by the Ethics Board of the Medical Faculty, Ludwig-Maximilians-University Munich (17-155). The improvement project and accompanying surveys were communicated via intranet, e-mail, and in team meetings of the respective units and departments. All surveyed professionals signed a letter of informed consent prior to participation.

Reporting of our findings adheres to publication guidelines for quality improvement reporting (revised Standards for Quality Improvement Reporting Excellence, SQUIRE 2.0) [30].

### OR-PICU handover setting and study sample at baseline and follow-up

Regular staffing of handovers includes anesthetists, occasionally supported by an anesthesia nurse, main surgeon or surgical assistant; as well as on the receiving end, a PICU physician and a PICU nurse. In this particular study setting, respiratory therapists are not involved in PICU handovers.

We applied a convenience sampling approach. OR-PICU transfers of intubated as well as non-intubated postoperative pediatric patients after surgical interventions were surveyed. We solely assessed handovers during day shifts (between 8 am and 6 pm). All OR and PICU physicians and nurses (with > 1 month organizational tenure) being involved in surveyed patient transfers were eligible.

### Data collection procedure at baseline and follow-up

During each surveyed handover, one trained expert observer was present on site. Altogether, three observers conducted the assessments. All had experience in clinical work and received training on the tools prior to commencement of data collections and familiarized with the handover procedure and setting beforehand. Before each wave, pilot training as well as pairwise observations were conducted to achieve familiarity as well as to establish consistency and inter-rater reliability. After the end of observed OR-PICU handovers, all present professionals were asked to fill out and return a short paper-based and anonymous survey, for measures see below.

### Baseline and follow-up measures

#### Expert-observation of OR-PICU handover performance

The observer rated performance using a locally modified but established standardized assessment on whether the action was performed correctly, or if the information was communicated clearly [16, 24]. Five distinct dimensions were of interest:

1. *Preparation of setup* is a list of items that captures potential errors in handover preparation, equipment, and technology handover prior to actual information handover (8 items; i.e., monitor and alarms not set, messy or confusing lines).
2. *Team engagement* assessed staff’s presence and consistent attentiveness (2 items, “all staff is attentive,” “all staff is concurrently present throughout the handover”).
3. *Information handover* includes items with essential pieces of information that should be explicitly mentioned during the handover or ensuing discussion. Three different professional content domains were considered:

-
  -  patient handover (7 items; e.g., name, age),
  - information provided by anesthesia (8 items; e.g., current status, intraoperative complications),
  - as well as information provided by surgeon (8 items; e.g., blood loss, antibiotics).

#### Expert- and staff-rated OR-PICU handover team performance

*Multi-professional team performance* during handovers is rated to evaluate post-operative handovers of multidisciplinary clinical teams [16]. It is an observational tool with behavioral markers to evaluate teamwork based on six different teamwork characteristics: (1) leadership (e.g., clearly defined team leader, good time management), (2) teamwork (e.g., good coordination, mutually supportive, assertive), (3) cooperation and resource management (e.g., performance of designated tasks for each member’s role, plans made prior to action), (4) communication and interaction (e.g., clear communication with team leader as hub), (5) workspace and equipment (e.g., appropriate equipment available when needed, correct operation of equipment, functionality checked), and (6) situation awareness (e.g., monitors visible, monitoring information gathered, recognition of patient state). Observer rated each teamwork characteristic on a 5-point Likert scale (1 = very poor; 5 = very good) [16].

Additionally, OR-PICU handover staff was surveyed on perceived team performance. After each observed handover, all participating professions were asked to fill out a survey identical to the observer survey. All six teamwork dimension items were provided with a short definition and rated on a five-point Likert scale (1 = very poor; 5 = very good) [16].

#### OR-PICU staff-rated mental workload

Participants also filled out a short survey on perceived mental workload during the handover task. Specifically, we measured the following three outcomes:

1. *Mental demands* were measured with one item adapted from the NASA-TLX questionnaire [31], “how high were the mental demands during the handover?” (scale: 0 = “very low,” 5 = “very high”);
2.  *Perceived disruptions* (*1* item, source: NASA-TLX) “how disruptive was the environment during the handover?” (0 = “very low,” 5 = “very high”);
3. *Perceived stress* was measured with a 6-item scale that quantifies the cognitive, emotional, and physical aspects of work stress [32]. It consists of six statements like, feeling calm (reversely coded), tense, or upset (response scale: 1 = “no, not at all” to 4 = “yes, completely”).

#### PICU-rated comprehensiveness of relevant handover information

Afterwards, the PICU physician managing the patient was surveyed concerning 9-key patient information items deemed essential for ensuing PICU care: e.g., list of current medication intake, ESBL/MRSA status, catecholamine doses, and instructions for post-operative care (all answered with yes/no/not applicable).

#### Qualitative process evaluation: interviews with OR and PICU professionals

To obtain information on the actual state of the implementation process, contextual factors, and effectiveness, we conducted interviews at follow-up with clinicians of all involved professions. The open interview guideline was derived from the literature and contained four major domains of interest [28, 33].

1. Review of current state and recent months of the intervention on improvement in day-to-day OR-PICU handovers;
2. Experiences of key facilitators and barriers to implementation and sustainability of intervention;
3. Subjective evaluation of specific contextual factors (i.e., patient safety culture, teamwork, leadership support, COVID-19-related influences,
4. Other experiences related to the intervention and its ensuing changes, comments, and suggestions.

After a convenience sampling approach, eight clinicians were interviewed by a member of the study team using a semi-standardized guideline on their evaluation and experiences of the intervention (with a mean duration of ca. 20 min).

### Statistical analyses

First, we computed means and standard deviations for quantitative outcomes for baseline and follow-up assessments, respectively. Provider-ratings were aggregated to mean scores at the level of each handover. Secondly, interrupted-time-series analysis was deployed to estimate the mean level change over time [34]. Data was analyzed with segmented regression analysis with 61 available data points, i.e., 31 handovers at baseline and 30 at follow-up. Autoregressive integrated moving average (ARIMA) models were estimated. All quantitative analyses were conducted with SPSS 29 (IBM, Chicago). To facilitate visual inspection of process improvement data over time, we additionally provide *p*-charts for all quantitative outcomes, respectively (see supplementary material). All eight interviews were analyzed by two reviewers with general inductive content analysis to identify key information on the four major domains of interest (see above) [35].

## Results

### Intervention

In course of the PDSA meetings, the intervention aimed for two areas of improvement: (1) a standardization of OR-PICU patient handovers was collaboratively defined into a standard operating procedure describing the individual steps of preparation (e.g., time line), intra professional handover tasks (e.g., physician-led handover process), and patient information transfer. (2) Additionally, a checklist for comprehensive information transfer was developed for standardized and comprehensive information transfer and structuring key patient information (i.e., patient, preoperative, anesthesia, operative, and postoperative details and essential laboratory values). A detailed description of the intervention, process, and adaption measures are described elsewhere [36].

In course of overall 11 inter professional and participatory meetings, solutions were developed, piloted, and internally evaluated; adjustments were considered and applied. Initially, five meetings were planned over the course of 5 months (Oct. 2017–Jan. 2018). Eventually, the meetings spanned from June 2018 to Oct. 2020.

### Effect evaluation

Overall, we surveyed 31 OR-PICU handovers at baseline (8 months observational period) and 30 at follow-up (12 months). At baseline, 37 OR-PICU patient handovers occurred, with 31 being eventually captured (83.8%). Across all included handovers, we approached afterwards 137 professionals. We obtained 111 surveys in return (81% response). Professionals were 26 ICU nurses (23.4%), 30 OR-anesthetists (27.0%), 4 surgeons (3.6%), 31 ICU physicians (27.9%), and 13 OR-anesthesia nurses (11.7%); 7 with missing (6.3%). At follow-up, we received 110 completed questionnaires with the following distribution across professions: 23 PICU nurses (20.9%), 33 OR anesthetists (30%), 12 surgeons (10.9%), 31 PICU physicians (28.2%), and 11 OR anesthesia nurses (10%).

### Outcome I: observer-rated OR-PICU handover performance over time

Concerning our quantitative outcome measures, we first determined mean percentages of expert observed handover performance and comprehensiveness of communicated information. Table 1 reports the mean values for baseline and follow-up as well as test for mean changes over time.

**Table 1:** Observer-rated OR-PICU handover performance over time (all in % of correct items)

	**% of observed items**			
	**Baseline**	**Follow-up**	**Significance testing (ARIMA)**	
**Outcomes**	Mean (95% CI min, max)		*B* (SE), *t* (*p*)	Goodness of fit (*R*^2^)
Preparation of handover setup	79.3 (71.0, 87.7)	98.6 (96.4, 100)	**19.1 (4.9), 3.9 (< .01)**	0.27
Team engagement	50.0 (38.4, 61.6)	81.7 (71.3, 92.0)	**31.6 (6.7), 4.7 (< .01)**	0.24
Information handover: patient	66.7 (56.9, 76.5)	79.3 (70.5, 88.1)	11.8 (8.5), 1.4 (.2)	0.11
Information handover: anesthesia	78.6 (73.1, 84.0)	87.3 (83.7, 90.9)	**8.5 (3.8), 2,3 (< .01)**	0.13
Information handover: surgeons	36.0 (26.5, 45.5)	31.7 (17.7, 45.7)	− 4.6 (6.9), − 0.7 (.5)	0.04

baseline n = 31 observations, follow-up n = 30, mean % of correct items

*CI* confidence interval, *ARIMA* autoregressive integrated moving average, *B* effect estimate for level change between baseline and follow-up, *p* significance, bold if *p* < .05

We observed significant improvements in three domains: preparation of team setup and patient was significantly improved at follow-up as well as enhanced team engagement and more comprehensive information transfer by the anesthesia sub-team (cf., Table 1).

### Outcome II: expert- and staff-rated OR-PICU handover team performance

Next, we analyzed for mean levels of expert and provider self-reported team performance as well for potential mean changes over time. Table 2 reports respective results.

**Table 2:** Observer and self-report team performance over time

	**Baseline**	**Follow-up**	**Significance testing (ARIMA)**	
**Outcomes**	Mean (95% CI min, max)		*B* (SE), *t* (*p*)	Goodness of fit (*R*^2^)
**Expert-rated team performance**				
Leadership	3.2 (2.9, 3.4)	4.5 (4.2, 4.8)	**1.3 (0.2), 7.1 (< .01)**	0.45
Teamwork	3.7 (3.5, 3.9)	4.6 (4.3, 4.8)	**0.9 (0.2), 5.4 (< .01)**	0.30
Cooperation	3.3 (3.0, 3.6)	4.6 (4.3, 4.9)	**1.3 (0.2), 7.2 (< .01)**	0.45
Communication	3.4 (3.1, 3.6)	4.4 (4.1, 4.7)	**1.1 (0.2), 4.7 (< .01)**	0.31
Workspace	3.2 (2.9, 3.5)	4.6 (4.3, 4.8)	**1.4 (0.2), 6.7 (< .01)**	0.44
Situation awareness	3.4 (3.1, 3.6)	4.8 (4.6, 5.0)	**1.4 (0.2), 9.6 (< .01)**	0.59
**Provider self-rated team performance**				
Leadership	3.8 (3.7, 4.0)	3.7 (3.5, 4.0)	− 0.1 (0.1), − 1.0 (.3)	0.09
Teamwork	4.1 (4.0, 4.3)	4.1 (3.9, 4.3)	0.0 (0.1), − 0.1 (.9)	0.08
Cooperation	3.7 (3.5, 3.9)	3.8 (3.6, 4.0)	0.1 (0.1), 1.3 (.2)	0.05
Communication	4.0 (3.8, 4.1)	3.9 (3.6, 4.1)	− 0.1 (0.1), − 0.7 (.5)	0.04
Workspace	3.9 (3.7, 4.1)	4.0 (3.8, 4.1)	0.1 (0.1), 0.6 (.6)	0.01
Situation awareness	3.9 (3.8, 4.1)	4.0 (3.8, 4.2)	0.1 (0.1), 0.7 (.5)	0.03

baseline *n* = 31 observations, follow-up *n* = 30, scale range for team performance outcomes: 1 = very poor, 5 = very good

*CI* confidence interval, *ARIMA* autoregressive integrated moving average, *B* effect estimate for level change between baseline and follow-up, *p* significance, bold if *p* < .05

At baseline, provider ratings were lower than expert ratings. Between baseline and follow-up, we observed a consistent improvement in all team performance measures based on expert observers’ ratings (cf., Table 2). In contrast, identical measures being reported by providers were not significantly different between both waves.

### Outcome III: OR-PICU staff-rated mental workload over time

Additionally, staff rated their mental workload after each handover. Table 3 displays the mean scores for baseline and follow-up assessments.

**Table 3:** OR-PICU staff-rated mental workload at baseline and follow-up

	**Baseline**	**Follow-up**	**Significance testing (ARIMA)**	
**Outcomes**	Mean (95% CI min, max)		*B* (SE), *t* (*p*)	Goodness of fit (*R*^2^)
Mental demands	3.2 (3.0, 3.4)	2.9 (2.6, 3.1)	− 0.30 (0.17), − 1.78 (.08)	0.06
Perceived disruptions	2.2 (1.9, 2.6)	2.1 (1.7, 2.5)	− 0.16 (0.27), − 0.59 (.56)	0.01
Perceived stress	1.6 (1.5, 1.7)	1.7 (1.6, 1.8)	0.04 (0.08), 0.55 (.58)	0.01

baseline *n* = 31 observations, follow-up *n* = 30, mean staff ratings

*CI* confidence interval, *ARIMA* autoregressive integrated moving average, *B* effect estimate for level change between baseline and follow-up, *p* significance, bold if *p* < .05

Overall, all provider-reported mental workload measures were in a fairly low to medium range. We did not observe a significant change over time in all three outcomes. Yet, perceived mental demands decreased substantially over time but did not achieve the prior established significance level.

### Outcome IV: comprehensiveness of PICU patient information

Concerning our last outcome, we identified a significant increase in completeness of post-operative patient information reported by admitting PICU physician after the intervention (cf. Table 4).

**Table 4:** Comprehensiveness of post-operative patient information reported by admitting PICU physicians over time

	**Baseline**	**Follow-up**	**Significance testing (ARIMA)**	
**Outcome**	Mean (95% CI min, max)		*B* (SE), *t* (*p*)	Goodness of fit (*R*^2^)
Comprehensiveness of post-operative patient information reported by admitting PICU physician (in %)	65.9 (57.6, 74.2)	76.2 (68.9, 83.4)	**10.2 (5.0), 2.0 (.048)**	0.06

baseline *n* = 31 observations, follow-up *n* = 30, mean % of correct items

*CI* confidence interval, *ARIMA* autoregressive integrated moving average, *B* effect estimate for level change between baseline and follow-up, *p* significance, bold if *p* < .05

### Interview information on implementation process, obstacles, and remaining challenges

Our qualitative analyses and results drawing upon the interviews with clinicians and nurses were clustered according to three pre-defined major topics of interests (see also above).

The first topic referred to the current state of the intervention and perceived changes in everyday OR-PICU handover practice. All interviewed professionals reported being aware of the intervention as well as being well informed about the improvement measures, i.e., introducing standardize postoperative patient handovers and concurrent implementation of checklists.

Secondly, concerning key facilitators and barriers for implementation and sustainability of the intervention, anesthetists reported greater familiarity with standardized handover processes stemming from other handover settings in the clinic that facilitated acceptance and adoption. Obstacle factors included frequent interruptions during handovers, long duration, and overall time pressure. As observed during post-implementation, a remaining hindering issue was the frequent absences of pediatric surgeons during handovers. Interviewees deemed it affecting fidelity and sustainability of revised OR-PICU handover practices. Pediatric surgeons acknowledged importance of being present, yet, they reported long waiting times until actual execution of postsurgical handovers, need for rest and breaks between interventions, and as well as overlaps with subsequent operations. Foremost, they experienced concurrent demands and additional obligations to draft a surgical report immediately after the operation (i.e., due to limited space and poor environmental conditions in the OR, they often preferred documentation away in their non-disturbing back offices). In their experience, these multiple postsurgical demands made it often inefficient to attend handovers. They often still favored individual surgical handovers on PICU, being more feasible and in line with workflow.

Thirdly, concerning subjective appraisal of specific contextual factors (e.g., patient safety culture, teamwork, leadership support, COVID-19-related influences), various issues were reported: participants stated that patient safety was always of prior attention; also in before the intervention, yet, the handover improvement was deemed to mitigate loss of key patient information as well as better structure of information transfer. Whereas, physicians perceived inter professional teamwork to be unchanged on a high level; nursing professionals reported they had more time to actively listen during patient handovers since ensuring that the patient’s well-being had been established prior to the exchange of information. All interviewees noted that communication among team members was improved. Concerning involvement and support from superiors, answers varied greatly: some felt sufficient involvement in the course of development and implementation, other reported low leadership support and feedback.

Further facilitators were small size of inter professional teams with efficient exchange in information during the discussion as well as high motivation and interest in the intervention. COVID-19 pandemic-related challenges were staff shortages, fewer operations, and frequent changes in medical personnel. Further barriers were reliable announcements when the handovers will take place eventually as well as perceived uncertainties concerning the levels of medical experience and knowledge of terminology by the receiving team members when exchanging complex patient information. Others mentioned insufficient training and instructions to staff members that joined after the actual intervention.

## Discussion

Patient handovers from the OR to PICU can be error-prone and carry potential for patient harm. Bundle interventions were proposed to improve post-operative handover practices [8, 22, 26, 27, 37]. We report implementation, participative redesign, and prospective effect evaluation of a standardized handover procedure intervention for OR-PICU patient transitions with a tool for structuring key patient information. Checklists are an effective tool for transfer of safety-relevant information [11]. We applied an interrupted time-series design and a mixed-methods evaluation. This study is to the best of our knowledge the first in pediatric care that utilized multiple evaluation sources such as expert observation, provider reports, and PICU physicians’ reports in pediatric care. Our results provide an inconsistent picture concerning intervention’s long-term effectiveness on handover performance, teamwork, and provider workload and stress. Yet, our findings contribute in various ways to the current evidence base on efficient and safe handover practices in pediatric care.

First, we observed changes in observer-rated OR-PICU handover performance in three out of five outcomes with significant improvements in team’s preparations of the handover, in engagement during the handover (i.e., higher attentiveness and presence in course of the handover), as well as for comprehensiveness of intraoperative information provided by the anesthesia sub-team. This increase can be attributed to our standardized handover practice intervention with higher efficiency between handoff and receiving professionals as well as higher attendance for handover duration [14, 38, 39]. Concerning the differences between the professions, we post hoc assume that adherence to protocol and checklist items as well as thoroughness of execution may have been different across professions [40]. Notwithstanding our inconsistent results on handover team performance, our empirical, prospective study contributes to the limited research base on teamwork processes underlying clinical handoffs in real-world pediatric care settings [16].

Secondly, we identified improvements in expert observed teamwork. Yet, those increases were in contrast to provider self-ratings: provider evaluations did not change over time. Expert-based observations of in situ care practices through trained observers have the potential to capture complexities of clinical practice and provide valuable insights into the performance and safety of patient care [41]. Yet, several explanations may apply post-hoc to our mixed observations: at baseline, provider ratings were on a high level with limited potential to increase; whereas, expert-baseline values were low to medium. It has been argued that positive provider attitudes are not necessarily accompanied by appropriate behaviors [42]. Moreover, in course of long intervention period, OR and PICU professionals may have shifted their expectations and subjective standards concerning effective teamwork behaviors such that real-world improvements were not noted afterwards. Another explanation may be due to the ongoing presence of external observers on site, professionals may have been displayed better team behaviors. Although we sought to establish reliability and consistency over time, we cannot exclude observer effects such that the observer at post implementation follow up may have been biased concerning the study aims.

Third, concerning OR-PICU staff-rated mental workload over time, we did not observe significant changes. Nonetheless, we noted a trend toward lower mental demands at follow-up (cf., Table 3). It has been argued that checklists serve as cognitive aids with particular effects for attentional focus and memory. Moreover, checklist use may alleviate mental demands and facilitate shift of attentional focus away from task, process, and team coordination to ensure all relevant patient information is conveyed in OR-PICU handoff [43]. Our results suggest that our intervention may facilitate mental demands in handover practice and contributing to ease cognitive overload and to facilitate comprehension of the handover situation and task demands [12, 23]. Concerning perceived levels of disruptions during the handover, it needs to be acknowledged that no particular measures were undertaken, to mitigate external interruption sources while handovers take place. Previous investigations showed that during postsurgical handovers, distractions and interruptions are common with potential to mitigate teamwork and information transfer [7, 12, 24, 44].

Fourth, we measured a significant higher comprehensiveness of key patient information being reported by receiving ICU physicians: an average increase of 10% from about 66% to 76%. Although we acknowledge that this is a self-reported and proxy indicator for actual clinical patient status and care outcomes, our results suggest that fewer omissions of essential patient information were made after implementation of a standardized handover process and checklist [45]. We acknowledge that after the intervention a substantial portion of essential patient information had not been effectively transmitted (i.e., an average of 24%). Ideally, receiver’s comprehension of the patient should be optimal for taking over care after handover. Further measures beyond standardization and checklist-based solutions need locally to be taken, to establish handover communication and conversation practices that safeguard sustainable transmission of essential patient information [8, 46].

Fifth, our qualitative analyses revealed multiple process factors that facilitated as well as hindered the implementation process of the intervention. In line with recommendations, we sought to establish a short-term stepwise PDSA-based improvement process with all OR and PICU professions [39]. Team engagement was perceived positive as an opportunity to adapt proposed solutions to local needs and intra professional requirements [22]. Yet, the development and pilot implementation as well as concurrent readjustments were longer than expected. Additionally, we faced substantial challenges to align all workflow requirements of all professions, especially alignment and inclusion of pediatric surgeons into a joint and concurrent handover process. After the intervention, various hindering system, organizational, and environmental conditions remained that favored idiosyncratic handover practices of pediatric surgeons. Potential remedies that might improve presence were discussed for future PDSA cycles (e.g., individual alerts shortly before handover starts, design for undisturbed workstations within the OR) and need to be tested during future improvement efforts. Yet, further solutions on-site and re-design need to take account of system- and environment-based constraints (i.e., poor OR layout, busy desktop workstations), intra- and inter professional workflows, patient care needs, safety and performance, as well as opportunities for physician respite and recovery [47].

Finally, in all quantitative outcome measures, we observed a substantial variability across all observations. In everyday clinical handover practice, process deviations are common and occur across all moments of patient’s surgical pathway [48]. Although we sought to utilize a time-series approach that accounts for variability, alternative attempts are warranted in the future that scrutinize variability changes over time due to alternated handover practices.

### Limitations

Our findings should be interpreted in the light of various limitations. The intervention was conducted in one academic pediatric hospital without a control group. All participants were informed about the intervention and concurrent evaluation. Since sometimes very complex cases are treated, results may therefore not be generalizable. One strength was to establish various sources for evaluating the effect of the intervention and utilizing combined assessments [49]. We acknowledge that inconsistencies occurred and that potential role-related differences in teamwork assessments may have not been captured, i.e., varying evaluations between nursing and physicians. Real-time and external expert observations on site are valuable, yet need resources such as flexibility, availability, and training. Although expert-observers were trained and tested for reliability, their background outside of pediatric medicine may influence how dynamic handover on site is tracked and evaluated [49]. Nonetheless, clinicians’ self-ratings may also encompass various sources of bias with individual notions what specific handover behaviors contribute effectively to everyday patient transfers [50, 51]. Project progress was delayed due to COVID-19 related burdens; various meetings were postponed as well as a number of surgical procedures were reduced for several months. Further barriers included local surgeons being not consistently able to reliably participate in the process. Other potential biases might be attributed to non-participation in surveys, selection in observed handovers, limited sample sizes, and secular trends that may have concurrently contributed to improved handovers practices.

Finally, our study did not measure if our intervention was associated with improved patient outcomes. Although our onsite assessment on PICU for comprehensiveness of essential patient information may be perceived as a surrogate for patient outcome measure, future investigations in this field should test for patient outcomes such as post-operative complications, inappropriate treatments, or morbidity outcomes. To this end, involvement of patients and children’s families in improvement practices, design, and outcome evaluations is warranted [18, 37].

### Implications for clinical practice and research

Concerning clinical practice, context-sensitive, and effective interventions are necessary that improve patient handovers in pediatric care. Handover tasks in everyday clinical practice encompass cognitive, communicative, and social challenges that require caregivers to integrate knowledge and skills into a brief, time-limited, and highly constrained activity [6, 23]. Well-designed handover environments and thoughtfully created procedures facilitate mutual co-construction of the course, direction, and outcome of the handoff and help receiving caregiver to plan subsequent steps of care accordingly [46, 52]. Nonetheless, smooth and effective handovers cannot be exclusively achieved by checklist or standardization interventions [19, 53]. Participative process and workflow re-design approaches that address all work system-components and draw upon OR and PICU professionals’ by-in are necessary to establish smooth handover processes [43, 54].

Concerning future research, developing a thorough understanding of effective postoperative handovers with deduction of adequate and evidence-based improvement measures in every day clinical practice remains an ongoing challenge to improve pediatric patient safety [7]. Our evaluation and its observational approach were augmented with provider self-reports as a unique and valuable attempt to capture genuine characteristics of handover practices that are not accessible to observations, i.e., cognitive system factors [43]. Nonetheless, future attempts should seek for additional measures to capture and elicit professionals’ experiences in situ care complexities.

## Conclusions

Our prospective results showed that a stepwise, participative improvement project that introduced standardized communication through the use of handover checklists improved observed handover team performance, and PICU-physician reported information transfer. Engaging OR and PICU professionals in interventions to improve multidisciplinary team performance during post-operative handover practice is essential to improve transitional safety of pediatric patients after surgery.

### Supplementary Information

Below is the link to the electronic supplementary material.

Supplementary file1 (PDF 741 KB)

## Data Availability

Anonymized data can be obtained from first author upon reasonable request.

## Abbreviations

CI: Confidence interval
df: Degrees of freedom
M: Mean
NASA-TLX: NASA—task load index
PDSA: Plan-do-study-action
PICU: Pediatric intensive care unit
OR: Operating room
SD: Standard deviation
SQUIRE: Standards of quality improvement reporting excellence
STAI: State trait anxiety index
