# Supplementary material for: Teamwork and mental workload in postsurgical pediatric patient handovers: Prospective effect evaluation of an improvement intervention for OR-PICU patient transitions
Source: Eur J Pediatr. 2023 Oct 11;182(12):5637–47. doi: 10.1007/s00431-023-05241-4 (PMC10746584; doi:10.1007/s00431-023-05241-4)

Electronic Supplementary Material

Supplementary Table 1: p-Charts of observer-rated OR-PICU handover performance outcomes over time (all in % of correct items; grey line: interval of PDSA handover intervention implementation)

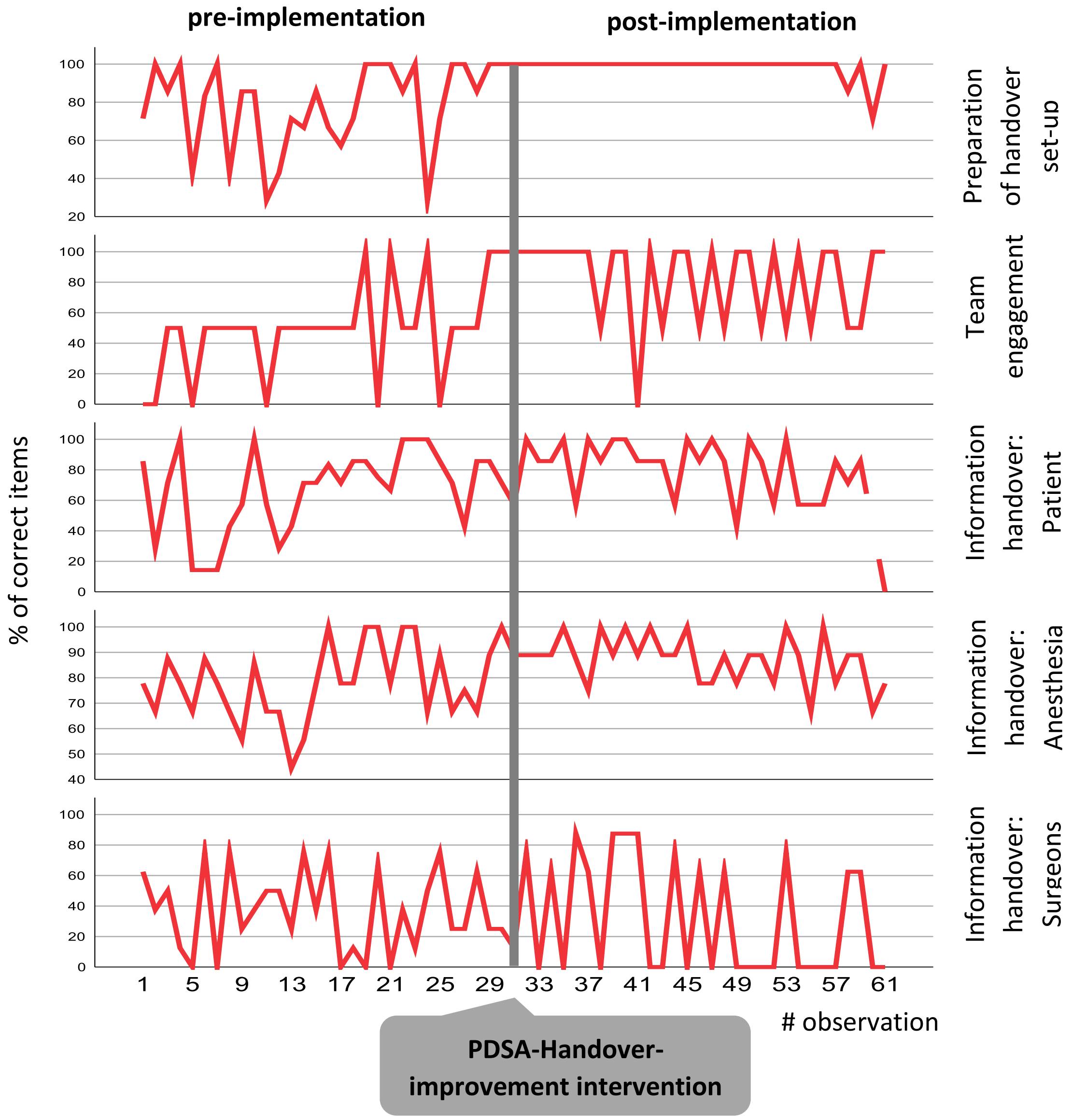

Supplementary Table 2: p-Charts of observer-rated team performance over time (mean values, grey line: interval of PDSA handover intervention implementation)

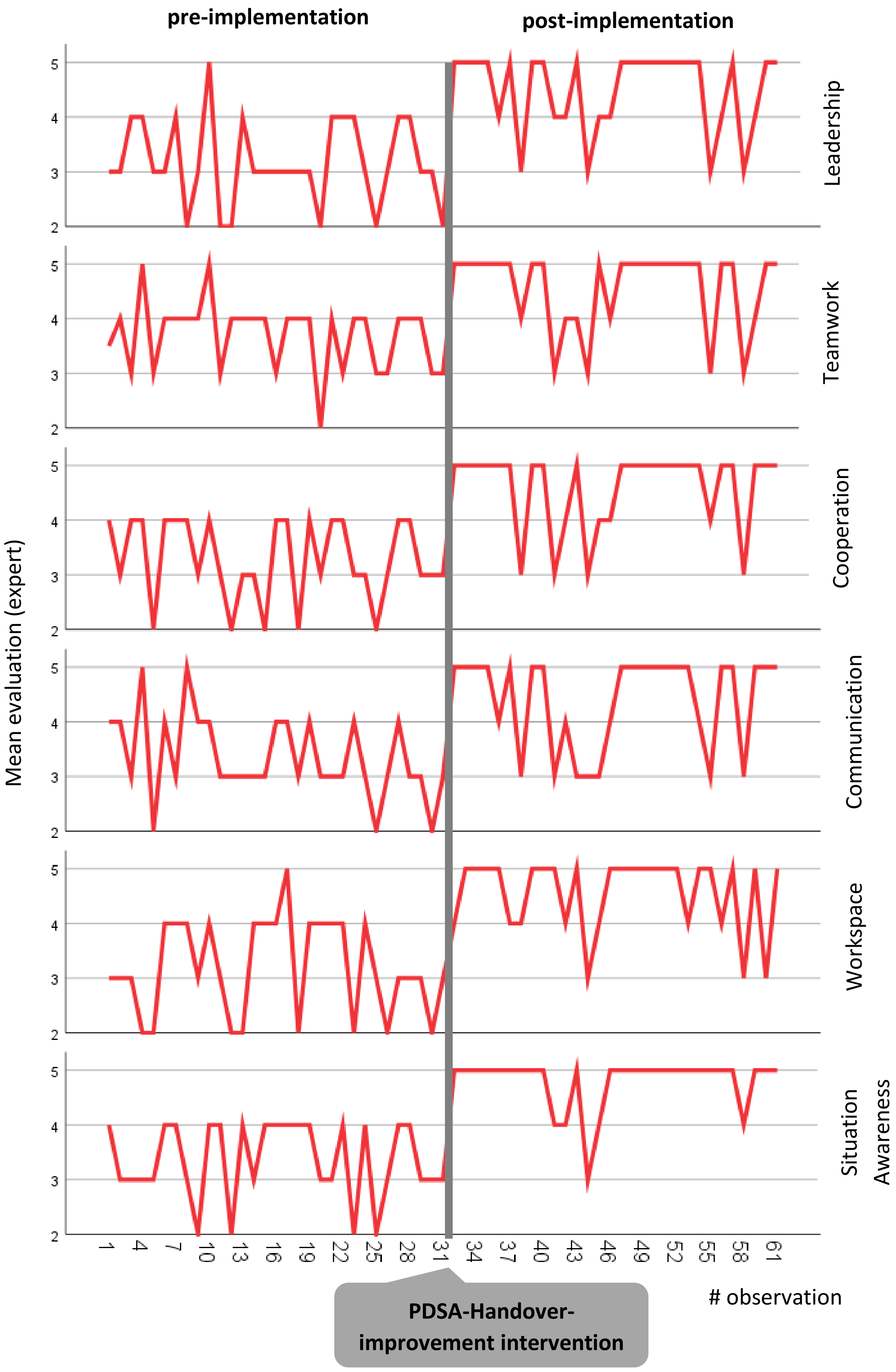

Supplementary Table 3: p-Charts of staff self-rated team performance over time (mean values, grey line: interval of PDSA handover intervention implementation)

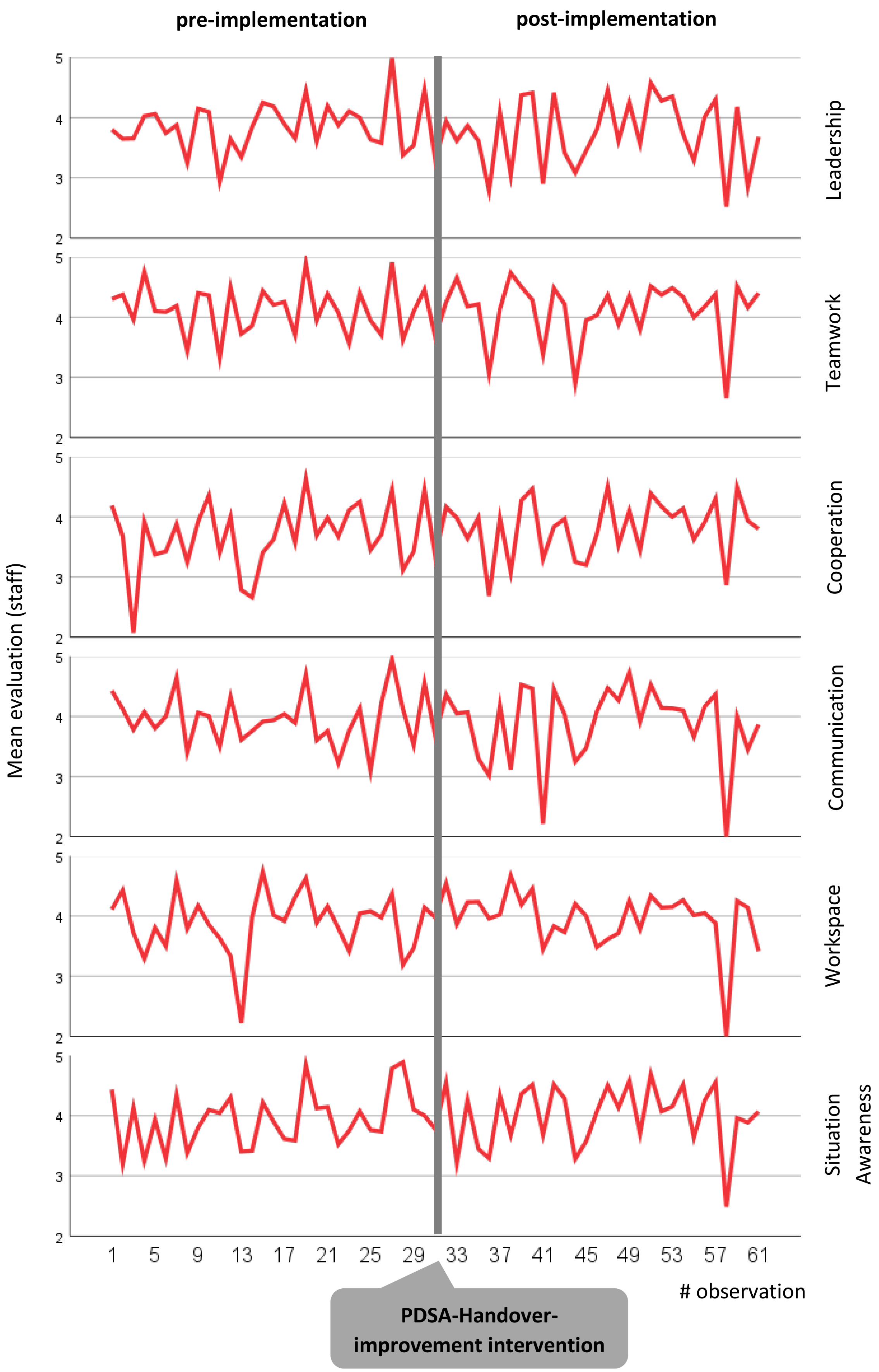

Supplementary Table 4: p-Charts of staff-rated mental workload outcomes over time (mean values, grey line: interval of PDSA handover intervention implementation)

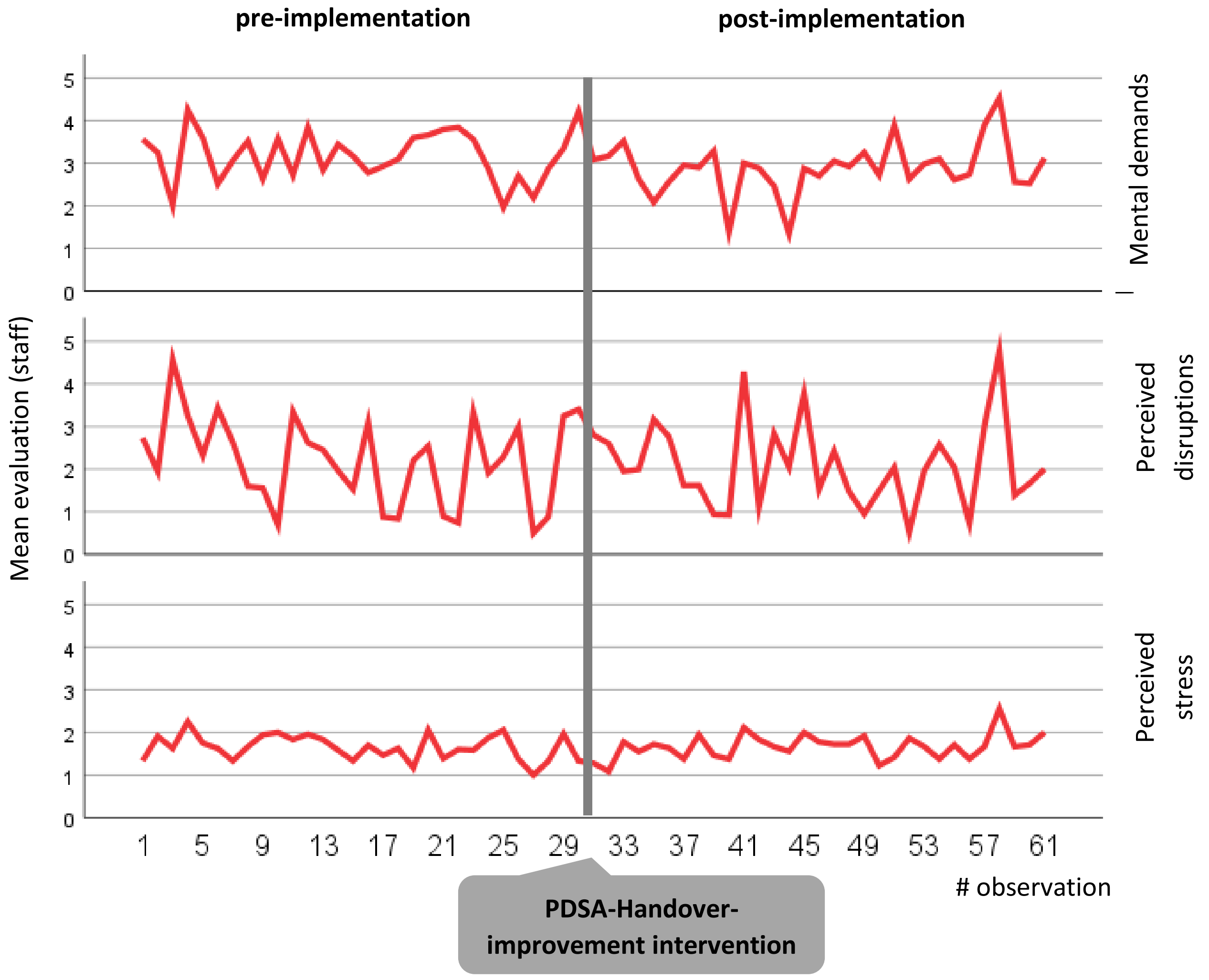

Supplementary Table 4: p-Charts of comprehensiveness of post-operative patient information reported by admitting PICU physicians over time (mean values of % of correct items, dotted line: intervention implementation)

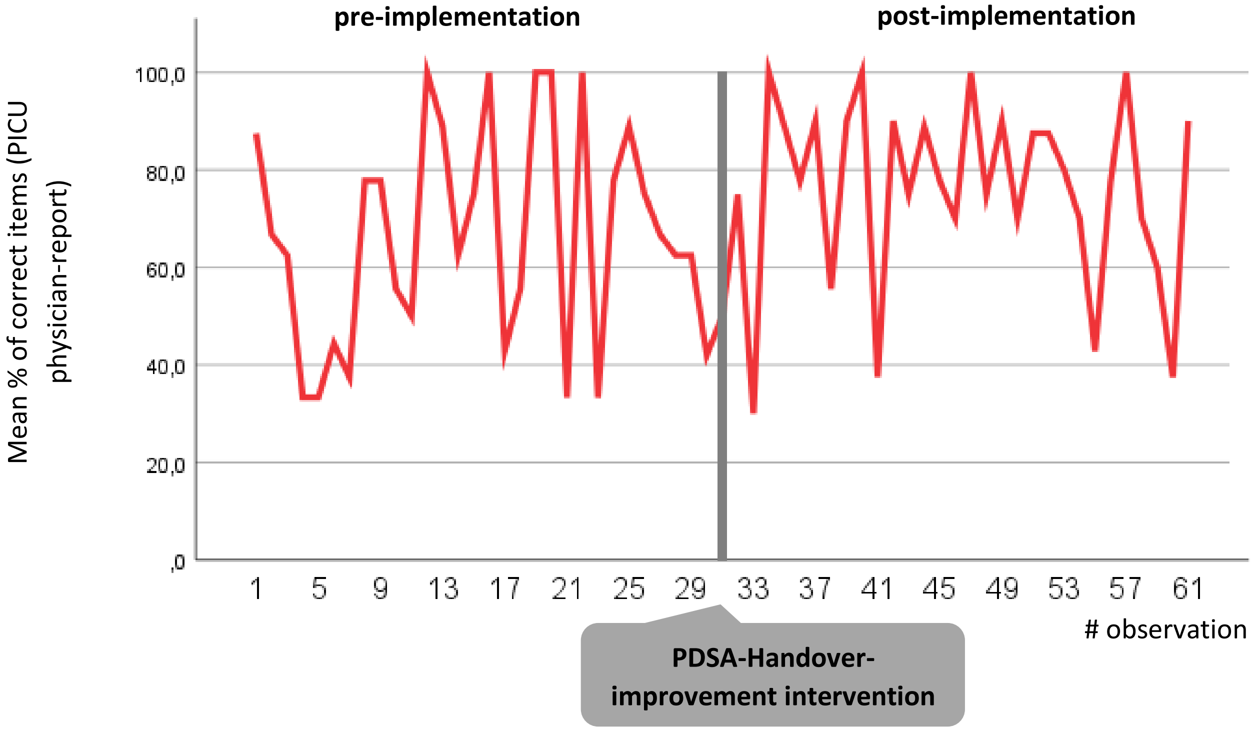

Supplement: Supplementary file 1 — Supplementary file1 (PDF 741 KB) [file 431_2023_5241_MOESM1_ESM.pdf]
